# Proteome analysis of schizophrenia patients Wernicke's area reveals an energy metabolism dysregulation

**DOI:** 10.1186/1471-244X-9-17

**Published:** 2009-04-30

**Authors:** Daniel Martins-de-Souza, Wagner F Gattaz, Andrea Schmitt, José C Novello, Sérgio Marangoni, Christoph W Turck, Emmanuel Dias-Neto

**Affiliations:** 1 Laboratório de Neurociências, Instituto de Psiquiatria, Faculdade de Medicina da USP, Rua Dr. Ovídio Pires de Campos, no 785, São Paulo, SP, CEP 05403-010, Brazil; 2 Max Planck Institute of Psychiatry, Kraepelinstrasse 2, D-80804, Munich, Germany; 3 Departamento de Bioquímica, Instituto de Biologia, UNICAMP, CEP 13083-970, Campinas, SP, Brazil; 4 Department of Psychiatry, Von Siebold Str. 5, University of Goettingen, 37075 Goettingen, Germany; 5 University of Texas, MD Anderson Cancer Center, 1515 Holcombe Blvd, 77030, Houston, Texas, USA

## Abstract

**Background:**

Schizophrenia is likely to be a consequence of DNA alterations that, together with environmental factors, will lead to protein expression differences and the ultimate establishment of the illness. The superior temporal gyrus is implicated in schizophrenia and executes functions such as the processing of speech, language skills and sound processing.

**Methods:**

We performed an individual comparative proteome analysis using two-dimensional gel electrophoresis of 9 schizophrenia and 6 healthy control patients' left posterior superior temporal gyrus (Wernicke's area – BA22p) identifying by mass spectrometry several protein expression alterations that could be related to the disease.

**Results:**

Our analysis revealed 11 downregulated and 14 upregulated proteins, most of them related to energy metabolism. Whereas many of the identified proteins have been previously implicated in schizophrenia, such as fructose-bisphosphate aldolase C, creatine kinase and neuron-specific enolase, new putative disease markers were also identified such as dihydrolipoyl dehydrogenase, tropomyosin 3, breast cancer metastasis-suppressor 1, heterogeneous nuclear ribonucleoproteins C1/C2 and phosphate carrier protein, mitochondrial precursor. Besides, the differential expression of peroxiredoxin 6 (PRDX6) and glial fibrillary acidic protein (GFAP) were confirmed by western blot in schizophrenia prefrontal cortex.

**Conclusion:**

Our data supports a dysregulation of energy metabolism in schizophrenia as well as suggests new markers that may contribute to a better understanding of this complex disease.

## Background

Several studies using global gene expression and proteomics analyses in different brain regions of schizophrenia (SCZ) patients have revealed dysfunctions in synaptogenesis and neural plasticity, energy metabolism, cytoskeleton assembly and oligodendrocyte metabolism [1-13].

One of the main clinical features in SCZ is the inappropriate use of language such as preservations, low verbal fluency, or absent-mindedness with use of incoherent language. They can be assumed under formal thought disorders and represent reliable diagnostic criteria for schizophrenia. As a complex and dynamic cognitive system that integrates multiple levels of linguistic and cognitive processing, language can be distinguished in micro- and macrolinguistic dimensions that are compromised in SCZ [reviewed in [14]]. The superior temporal gyrus (STG), which is part of the human temporal lobe, is the major brain area related to speech, language and communication. Injuries such as tumors, stroke or epilepsy affecting the STG may lead to disturbances of language function and hallucinations similar to those observed in SCZ symptoms [15]. The left STG (L-STG) gray matter was shown altered compared to the right STG as well as the brain symmetry in SCZ patients [16-19]. Interestingly, the volume of the L-STG cortex was negatively correlated with deficit symptoms [18] and was shown to be decreased to a larger extent in patients with poor-outcome compared to those with good outcomes [20]. Binding sites of the glutamatergic N-methyl-D-aspartic acid (NMDA) receptor have been reported to be increased in the STG to a greater extent on the left side in patients with predominantly negative symptoms [21,22]. The L-STG, specialized in language skills, is formed by the primary auditory cortex (Brodmann's Areas (BA) 41 and 42) and the Wernicke's area (WA), also described as the posterior region of BA22. WA is an important region for speech processing and language skills.

We performed comparative individual proteomic analyses of L-STG (WA or BA22p) tissues of 9 SCZ patients and 6 controls, using two-dimensional gel electrophoresis (2-DE), followed by MALDI-TOF/TOF (Matrix-Assisted Laser Desorption/Ionization/Time of Flight) mass spectrometry. The 25 regulated proteins identified here include novel as well as previously reported putative SCZ markers that may be involved in disease pathobiology.

## Methods

### Human Wernicke's area (WA)

*Post-mortem *brain samples from the left WA tissue (BA22p) were collected from 9 schizophrenia patients and 6 controls. Control subjects were free from psychiatry disorders, somatic diseases or brain tumors and had never been treated with antidepressant or antipsychotic medications. Brain samples were dissected by an experienced neuropathologist (median: 20.5 hours (min.7 hours – max.37 hours) after death) and deep-frozen immediately after collection.

All samples were obtained from the brain bank of the Central Institute of Mental Health (Mannheim, Germany). Controls were collected at the Institute of Neuropathology, Heidelberg University, and clinical records were collected from relatives and general practitioners. Patient samples were derived from in-patients of the Mental State Hospital Wiesloch, Germany. All cases and controls were German whites. All SCZ patients were long-term in-patients at the Mental State Hospital Wiesloch, Germany, and SCZ diagnosis was made *ante-mortem *by an experienced psychiatrist according to the DSM-IV criteria [23]. For each patient the antipsychotic treatment history was assessed by examining the medical charts and calculated in chlorpromazine equivalents (CPE), through the algorithm developed by Jahn and Mussgay [24] for typical neuroleptics and clozapine. The CPE of olanzapine was calculated using the mean doses described by Meltzer and Fatemi [25]. All patients and controls underwent neuropathologic characterization to rule out associated neurovascular or neurodegenerative disorders. The classification according to Braak was stage II or less for all subjects [26,27]. Patients and controls had no history of alcohol or drug abuse and severe physical illness. All assessments and *post-mortem *evaluations and procedures were previously approved by the ethics committee of the Faculty of Medicine of Heidelberg University, Germany. Detailed patient information is given in Additional file 1.

### Sample Preparation

Fifty milligrams of human WA were individually homogenized in 1.5 ml tubes with glass spheres in 200 μl of 7 M urea, 2 M thiourea, 4% CHAPS, 2% ASB-14 and 70 mM DTT buffer [28]. Samples were centrifuged for 10 min at 14,000 rpm and quantified [29]. Supernatants were used for 2-DE.

### Two-Dimensional Gel Electrophoresis

Using two-dimensional gel electrophoresis we performed an individual proteome analysis of 9 schizophrenia samples compared to a pool of 6 healthy control. The experiments were carried out as described by Martins-de-Souza et al. [28]. Briefly, 650 μg of protein from SCZ or control samples were applied to IPG gel strips with a nonlinear separation range of pH 3–10 prior the second dimension in 12.5% T acrylamide gels that were stained using a colloidal coomassie blue protocol.

All experiments were performed in triplicate. Only proteins that appeared to be differentially expressed in triplicate gels were considered as differentially regulated.

### Determination of protein expression differences and protein identification by peptide mass fingerprinting

2-DE gel images were used for spot detection and pI/MW calibration of WA samples using the ImageMaster 2D software, (GE Healthcare, Uppsala, Sweden). A total of thirty 2-DE profiles were analyzed, consisting of 27 gels from patients (9 patients evaluated individually in triplicates) plus three gels for the controls (triplicates of pooled controls). Volumes of all spots in SCZ and CTRL gels were determined and corresponding spots were matched for all 2-DE profiles. The spot volumes were analyzed by Kruskal-Wallis one-way analysis of variance aiming to reveal the statistically significant differences in the protein expression between the two groups. Protein spots with a mean n-fold change between SCZ and CTRL WA gels of +/- 1.8 were excised for mass spectrometry (MS) identification. False discovery rate (FDR) was calculated according to Storey, 2002 [30]. Protein identification by peptide mass fingerprinting was done as described by Martins-de-Souza et al. [31].

### Western Blot

For western blot analysis, proteins were extracted as described above. One-hundred μg of total protein from dorsolateral prefrontal cortex (DLPFC) were run on a 12% SDS minigel (Bio-Rad, Hercules, CA, USA) and the proteins were transferred to Immobilon PVDF membranes (Millipore, Bedford, MA) at 100 V for 1 h with cooling. Membranes were treated with 5% Carnation instant nonfat dry milk in TBS-T for 4 hours, rinsed in TBS-T 3 times for 20 minutes and incubated with anti-PRDX6 (Abcam, Cambridge, UK) or anti-GFAP antibodies (Abcam, Cambridge, UK) at a 1:1000 dilution in TBS-T overnight at 4°C. In the next day, membranes were washed with water and TBS-T for 15 min. Incubations with anti-c-MYC-peroxidase antibody (GE Healthcare, Uppsala, Sweden) were carried out for 40 min at room temperature, followed by membrane washing with water and TBS-T and incubation with ECL mixture (GE Healthcare, Uppsala, Sweden) for 1 min and ECL film exposure (GE Healthcare, Uppsala, Sweden). Films were scanned, and the band signals (optical densities) were assessed using QuantityOne software (Bio-Rad, Hercules, CA, USA).

### Protein classification

Differentially expressed proteins were classified according to their main biological processes as well as their molecular functions using Human Protein Reference Database (HPRD – ). Moreover, the WA differentially expressed proteins were classified according their biochemical pathways using KEGG analysis  through software "Blast2Go" .

## Results

**Figure 1:**
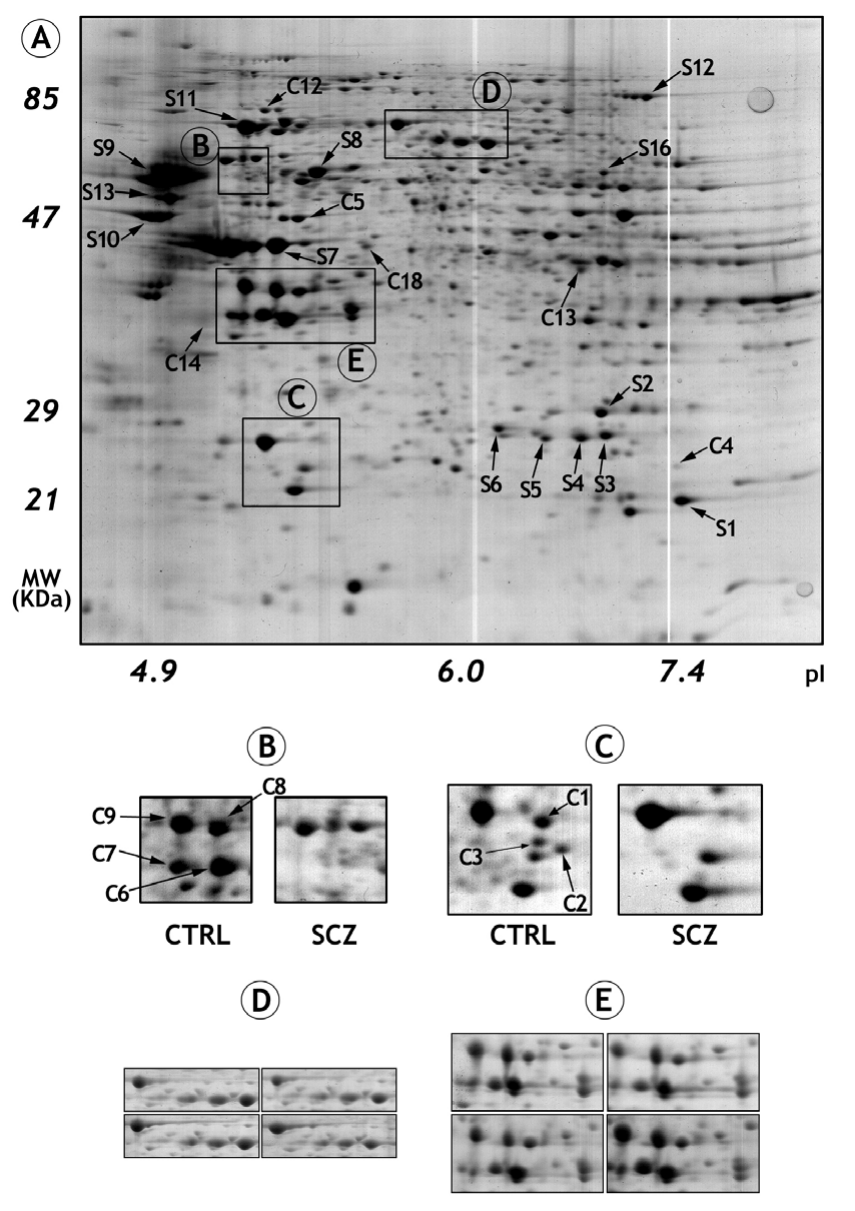
**Representative 2-DE profile of Wernicke's area from schizophrenia samples is presented in A**. The "S" means spot upregulation in SCZ and "C" spot upregulation in CTRL. Examples of proteins upregulated in CTRL brains are presented in the enlarged sections of the 2-DE profile (B and C). The reproducibility of the 2-DE profiles is demonstrated by the sections of four gels shown in D and E.

**Figure 2:**
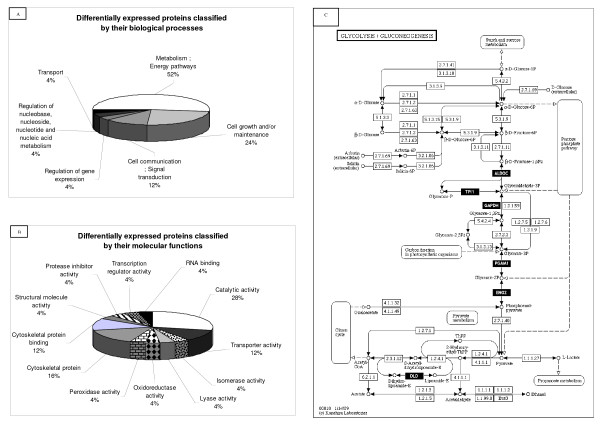
**(A) Biological processes and (B) molecular functions of differentially expressed WA proteins in SCZ**. (C) KEGG analysis showed that some of the differentially expressed enzymes identified here (ALDOC, TPI1, GAPDH, PGAM, ENO2 and DLD) belongs to the same glucose-related pathway.

On average, 669 protein spots were detected in SCZ 2-DE profiles whereas 605 protein spots were detected in CTRL 2-DE profiles (Figure 1A). There were 553 matched spots between the groups, representing 86.8% of the average number of spots. When individual SCZ gels and pool control gels were compared, 30 spots (~4,7%) were consistently identified with significant changes in relative abundance (> 1.4 fold difference, P < 0.05, Kruskal-Wallis) (Additional file 2) [31-38]. These 30 spots revealed 25 distinct proteins with apparent altered regulation (11 downregulated and 14 upregulated in SCZ samples; Figure 1). Twenty-three proteins were identified as single spots and two proteins were identified in multiple spots (spots C05, C06, C07, C10 and C08, C09; Figure 1). All 25 proteins were successfully identified by MALDI-TOF/TOF and grouped according to their biological processes and molecular function using the Human Protein Reference Database (HPRD – ) (Figure 2A and Figure 2B and Additional file 2). Equivalent areas of four distinct 2-DE gels are presented in the Figure 1D and 1E as an example of the reproducibility of the protein profiles.

## Discussion

Our findings support previous reports on altered protein expression in SCZ as well as suggest new targets that may be relevant for the pathobiology of this disease. The main pathways and proteins observed are discussed bellow.

### Energy metabolism dysregulation

Most proteins we identified as differentially expressed (52%) are enzymes involved the regulation of energy metabolism. Their altered levels can lead to an overall disturbance, and ultimately contribute to the establishment of pathological states [39,40]. In this regard, a recent report has suggested that brain evolution from monkeys to humans may have led to the appearance of SCZ due to changes affecting energy pathway genes [41]. The alteration of this pathway, specifically the glycolysis and gluconeogenesis pathways in the WA is strongly supported by our data (Figure 2C).

Enzymes with functions in glycolysis such as ALDOC (Aldolase C), TPI1 (Triosephosphate isomerase 1), GAPDH (Glyceraldehyde-3-phosphate dehydrogenase), PGAM1 (Phosphoglycerate mutase 1) and ENO2 (Enolase 2) were found to be differentially expressed in WA, and are very likely to affect the glucose metabolism in SCZ patients. Likewise a dysfunction in ATP metabolism can result from differentially expressed ATP synthase subunits, ATP6V1A (ATPase, H+ transporting, lysosomal 70 kDa, V1 subunit A) and ATP5A1 (ATP synthase subunit alpha, mitochondrial). The above-mentioned proteins, and in some cases their genes, have been previously found to be differentially expressed in SCZ (Additional file 2).

We also found the differential regulation of dihydropteridine reductase (QDPR upregulated: 2.25×), which has been previously found to be differentially regulated [7] in 10 SCZ patients compared to 10 paired controls. This enzyme is a key in the control of dopamine and serotonin synthesis [42].

**Figure 3:**
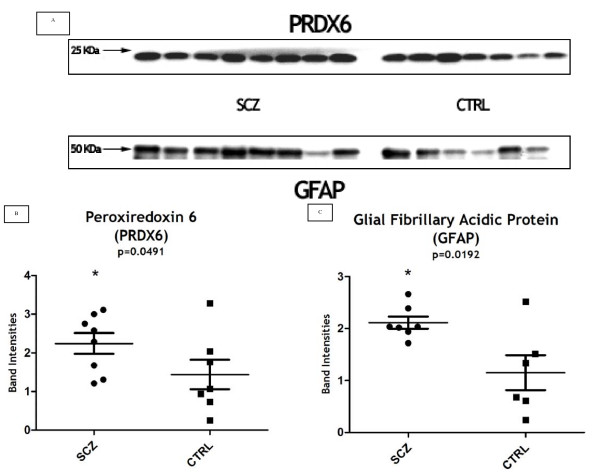
**(A) Western-blot profiles of PRDX6 and GFAP in the dorsolateral prefrontal cortex from SCZ and controls**. The intensity of the western-blot bands for both proteins and the statistical analysis by Mann-Whitney are presented in B and C.

In our analyses we also found two new energy-metabolism-related proteins to be regulated in SCZ, which may have important roles SCZ negative symptoms: dihydrolipoyl dehydrogenase (DLD: upregulated: 2.17×) and peroxiredoxin 6 (PRDX6 upregulated: 2.33×). DLD is a component enzyme of complexes such as pyruvate dehydrogenase, alpha-ketoglutarate dehydrogenase, and the branched-chain alpha-keto acide dehydrogenase which participate in cell redox processes. PRDX6 is a bifunctional enzyme with two active sites with distinct roles. This enzyme is involved in redox regulation protecting the cell against oxidative injury by reducing levels of H_2_O_2_, short chain organic fatty-acids and phospholipid hydroperoxides [43]. Oxidative stress that can lead to DNA damage, protein inactivation, altered gene expression and apoptotic events, probably starts in SCZ during neurodevelopment. The close connection of these mechanisms to neuronal plasticity suggests that an oxidative component may be relevant to disease pathogenesis [39,40,44]. Thus, it is plausible that the differential regulation of an oxidative protector like PRDX6 may contribute to pathogenesis. The phospholipase A2 (PLA2) activity of PRDX6 on the other hand is critical for the regulation of phospholipid turnover [43]. An accelerated PLA2-mediated phospholipid turnover was previously observed in SCZ frontal lobe [45] whereas an increased activity of Ca^2+^-dependent PLA2 may lead to a reduced brain dopaminergic activity [45,46]. The differential regulation of PRDX6 may accelerate the phospholipid turnover, leading to the described differential dopaminergic state. As both PRDX6 activities seem to be related to SCZ pathogenesis, we analyzed PRDX6 protein expression by western blot (WB) in the DLPFC of SCZ patients (Figure 3A). Again, a significant differential expression of PRDX6 was found (Figure 3B). Moreover, it is interesting to note that PRDX6 gene is localized in a chromosome loci (1q25.1) previously related with SCZ [47].

Although a correlation between the differential expression of glucose metabolism and oxidative phosphorylation enzymes has been recently described in SCZ [48], there is no consensus whether the protein expression alterations are causes or consequences of a pathological process. Also, the mitochondrial protein alterations found in SCZ could be consequences of the medications used by patients [32]. However, alterations in the glucose metabolism have been extensively reported as a central component of SCZ and are unlikely due to antipsychotic effects [41,49].

### Tubulin

Tubulins are involved in multiple activities, including mitosis, cytokinesis, and vesicular transport. Alterations of tubulin subunits have been described in SCZ [7,12,31,50] and were also found in WA in our study (Additional file 2). Importantly, possible roles of tubulin in SCZ have been suggested by Denarier et al. [51] who generated a mouse SCZ model by knocking out the stable tubulin-only polypeptide (STOP) gene, which is responsible for microtubule stabilization in neurons. STOP null mice have a normal brain anatomy but show synaptic function defects due to dysfunctional glutamatergic transmission [52]. Moreover, this model exhibits a hyperdopaminergic state in the limbic system [53].

### Other potential markers for schizophrenia

#### Glial Fibrillary Acidic Protein (GFAP)

Astrocyte abnormality is a recurrent finding in SCZ. Among other functions, astrocytes are responsible for the maintenance of glutamatergic transmission *via *the glutamate-glutamine cycle [54]. Glial fibrillary acidic protein (GFAP – upregulated: 2.31×) is the major intermediate filament (IF) protein of mature astrocytes. Modifications in the expression of this structural protein have been reported in SCZ (Additional file 1) and can lead to compromised synaptic functioning and behavior alterations [reviewed in [54]]. The over expression of GFAP, suggested by our 2-DE analysis was also evaluated and confirmed by western-blot in individual samples of SCZ-DLPFC (Figures 3A and 3C), reinforcing its potential role in SCZ.

#### Phosphatidylethanolamine-binding protein (PEBP1)

Phosphatidylethanolamine-binding protein 1 (PEBP1 – upregulated: 1.87×) has functions in membrane biogenesis and signaling mechanisms during cell growth and maturation. PEBP1 is a calpain substrate that has been implicated in processes that produce persistent changes in synaptic chemistry and structure [55]. A PEBP1 knockout mouse has been reported in 2007 [56], and revealed some interesting associations of this protein in the control of emotions and complex behavior responses. PEBP1 -/- mice suffer from a decline of the smell sense and reproduction without any obvious fertility defects. PEBP1 appears to be upregulated in the aggression and fear controlling amygdala. In a mouse model of Alzheimer's disease the loss of PEBP1 function was observed and behavioral tests revealed a learning deficit [57].

#### Breast cancer metastasis-suppressor 1 (BRMS1)

The BRMS1 gene product seems to act as a transcriptional regulator of diverse genes [58], and is capable to reduce the metastatic potential of human breast cancer and melanoma cell lines. This is the first description of the putative alteration of BRMS1 in SCZ or any other neuropsychiatric condition.

## Conclusion

It is important to mention that confounding factors such as other co-existing diseases, age, gender, or diet of the patients or *post-mortem *intervals and agonal states, can significantly impact brain proteome studies. For the particular set of samples analyzed in this study, the electrophoretic profile of proteins from individual samples from cases and controls suggests that the *post-mortem *intervals did not generate false proteome alterations in these particular analyses (data not shown). Moreover, we should note that all the SCZ samples used in the present work were obtained from patients that have been treated with antipsychotics. Distinct medications and distinct doses or treatment regimens can certainly impact the proteome studies presented here. We have calculated the false discovery rate (FDR) of the presented dataset using the Q-value software  developed by Storey 2002 [30]. FDR is a false positive calculation method that, instead of controlling the chance of any false positives (as Bonferroni), controls the expected proportion of false positives. The q-value given by FDR is the analogue of the p-value. Briefly, the q-value is a p-value of all the p-values simultaneously. The calculated FDR was 0.0153, suggesting that our data could have an error of 1.53%. The validation of the differentially expressed proteins, as we did for GFAP and PRDX6, would be an alternative to validate the markers and to skip possible errors.

As was found for other brain regions, our data suggest an overall energy metabolism dysregulation in WA of SCZ patients. Beasley et al. [32] suggested that many of the observed energy metabolism alterations could be consequences of medication. On the other hand Stone et al. [49] state that a dysfunctional energy metabolism is a central component of SCZ and not due to an antipsychotic effect. However, studies using samples from psychotropic drug naïve patients will hopefully help to resolve this issue.

Our findings are in line with a recent hypothesis of Khaitovich et al. [41] that states that SCZ may be a consequence of human brain development. Clustered genes that were positively selected during the evolution of the human brain were grouped into 22 functional categories, six of these (27,2%) included genes with functions related to energy metabolism, some of which previously shown to be related with SCZ. The concentration of metabolites affected by the corresponding proteins, including lactate, choline, and acetate were found to be different between SCZ and control brains. It is further suggested that human cognitive abilities are very sensitive towards alterations in metabolite levels and even slight brain energy metabolism dysregulations can lead to conditions that are hallmarks of SCZ.

The consistent individual identification of altered expression levels of the same proteins derived from patient specimens subjected to distinct therapeutic regimens support a potentially important role for these biomarkers in SCZ pathobiology. This includes PRDX6 and GFAP that were also found in DLPFC samples. Our data add to the importance of energy metabolism pathways for SCZ and have the potential to be translated to the clinic.

## Competing interests

The authors declare that they have no competing interests.

## Authors' contributions

This work is part of the post-doc project of DMS, who executed all the experiments and wrote the first manuscript draft. WFG and AS provided the brain samples, clinical data, and contributed to the manuscript writing and scientific discussions. SM and JCN provided the support for 2-DE analysis and mass spectrometry. CWT provided guidance for the mass spectrometry and western blot analysis and contributed to the manuscript writing. SM and EDN supervised this work and the manuscript writing. The study was conceived by DMS and EDN. All authors contributed to and have approved the final version of this manuscript.

## Pre-publication history

The pre-publication history for this paper can be accessed here:



## Supplementary Material

Additional file 1 **Table 1**. Patient and control clinical data. Using Mann-Whitney test, we found no significant differences between patients and controls for age (p = 0.5959), PMI (p = 0.2888) and pH (p = 0.7237). Click here for file

Additional file 2 **Table 2**. Proteins regulated in schizophrenia samples, classified according to their biological and molecular function. The accession numbers are from the Swiss-Prot database. Click here for file
