# Supplementary material for: Proteome analysis of schizophrenia patients Wernicke's area reveals an energy metabolism dysregulation
Source: BMC Psychiatry. 2009 Apr 30;9:17. doi: 10.1186/1471-244X-9-17 (PMC2684104; doi:10.1186/1471-244X-9-17)
Supplement: Additional file 1 — Table 1. Patient and control clinical data. Using Mann-Whitney test, we found no significant differences between patients and controls for age (p = 0.5959), PMI (p = 0.2888) and pH (p = 0.7237). [file 1471-244X-9-17-S1.doc]

***Table 1:*** Patient and control clinical data. Using Mann-Whitney test, we found no significant differences between patients and controls for age (p=0.5959), PMI (p=0.2888) and pH (p=0.7237).

| ***Sample ID*** | ***Case*** | ***Age (years)*** | ***Gender*** | ***PMI (hours)*** | ***pH***  ***values*** | ***Type of SCZ*** | ***Duration of Disease (years)*** | ***Duration of Medication (years)*** | ***atyptyp*** | ***CPE last dosis*** | ***CPE last ten years*** | ***Cause of Death*** | ***DSM IV*** | ***Age at Onset*** | ***Last Medication*** | ***Cigarettes*** | ***Alcohol*** | ***Hosp*** | ***ECT*** |
| --- | --- | --- | --- | --- | --- | --- | --- | --- | --- | --- | --- | --- | --- | --- | --- | --- | --- | --- | --- |
| 35/00 | SCZ | 64 | F | 23 | 6.6 | Residual, Chronic  Paranoid episodes | 41 | 40 | 2 | 54.5 | 4.6 | heart infarction | 295.6 | 24 | Zotepine 150 mg  Olanzapine 10 mg | 20/day | no | 5 | yes |
| 46/00 | SCZ | 63 | F | 31 | 6.8 | Residual, Chronic  Paranoid episodes | 40 | 30 | 3 | 75 | 1.8 | heart infarction | 295.6 | 24 | Olanzapine 15 mg | 30/day | no | 30 | yes |
| 48/00 | SCZ | 51 | M | 7 | 6.4 | Residual, Chronic  Paranoid episodes | 25 | 25 | 1 | 174 | 0.6 | heart infarction | 295.6 | 19 | Flupenthixol 15 mg | 30/day | no | 20 | no |
| 83/01 | SCZ | 71 | M | 28 | 6.4 | Residual, Chronic  Paranoid episodes | 40 | 35 | 1 | 782,4 | 10 | heart infarction | 295.6 | 30 | Haloperidol 32 mg, Pipamperone 40 mg | 40/day | no | 12 | no |
| 39/02 | SCZ | 43 | M | 18 | 6.9 | Residual, Chronic  Paranoid episodes | 22 | 20 | 2 | 464 | 2.6 | heart infarction | 295.6 | 20 | Zuclopethixol 40 mg, Valproate 1200 mg, Tiapride 300 mg | 0 | no | 13 | no |
| 75/02 | SCZ | 92 | F | 37 | 6.9 | Residual, Chronic  Paranoid episodes | 51 | 48 | 1 | 100 | 3.4 | pancreas-carcinoma | 295.6 | 41 | Prothipendyl 160 mg,  Perazine 100 mg | 0 | no | 51 | no |
| 13/00 | SCZ | 64 | F | 11 | 7.0 | Residual, Chronic  Paranoid episodes | 48 | 45 | 3 | 1536 | 7.7 | Pulmonary  insufficiency | 295.6 | 16 | Clozapine 500 mg  Haloperidol 4 mg | 0 | no | 21 | yes |
| 39/03 | SCZ | 77 | F | 32 | 6.8 | Residual, Chronic  Paranoid episodes | 49 | 48 | 2 | 2555 | 8.3 | Lung embolism | 295.6 | 28 | Clozapine 400 mg  Benperidol 25 mg | 0 | no | 48 | yes |
| 43/03 | SCZ | 76 | F | 17 | 7.1 | Residual, Chronic  Paranoid episodes | 49 | 47 | 1 | 300 | 4.9 | Cardio-pulmonary insuffiency | 295.6 | 27 | Perazine 300 mg | 0 | no | 30 | yes |
|  |  |  |  |  |  |  |  |  |  |  |  |  |  |  |  |  |  |  |  |
| 72/02 | Control | 79 | M | 24 | 6.4 |  |  |  |  |  |  | heart infarction |  |  |  | 0 | no |  |  |
| 02/02 | Control | 41 | M | 7 | 7.2 |  |  |  |  |  |  | heart infarction |  |  |  | 0 | no |  |  |
| 43/01 | Control | 91 | M | 16 | 6.8 |  |  |  |  |  |  | Cardio-pulmonary insufficiency |  |  |  | 0 | no |  |  |
| 51/02 | Control | 57 | M | 24 | 6.9 |  |  |  |  |  |  | heart infarction |  |  |  | 0 | no |  |  |
| 57/02 | Control | 53 | M | 18 | 7.0 |  |  |  |  |  |  | heart infarction |  |  |  | 0 | no |  |  |
| 59/02 | Control | 63 | M | 13 | 6.5 |  |  |  |  |  |  | heart infarction |  |  |  | 0 | no |  |  |

Abbreviations: PMI: *post-mortem* interval; atyptyp: duration of atypical treatment/duration of treatment with typical neuroleptics during lifetime; CPE: medication calculated in chlorpromazine equivalents (mg); CPE last ten years: the sum of medications during the last ten years in kg; Hosp: Hospitalization time in years; ECT: electroconvulsive therapy.
