# Supplementary material for: Proteome analysis of schizophrenia patients Wernicke's area reveals an energy metabolism dysregulation
Source: BMC Psychiatry. 2009 Apr 30;9:17. doi: 10.1186/1471-244X-9-17 (PMC2684104; doi:10.1186/1471-244X-9-17)
Supplement: Additional file 2 — Table 2. Proteins regulated in schizophrenia samples, classified according to their biological and molecular function. The accession numbers are from the Swiss-Prot database. [file 1471-244X-9-17-S2.doc]

***Table 2:*** Proteins regulated in schizophrenia samples, classified according to their biological and molecular function. The accession numbers are from the Swiss-Prot database.

| ***Biological Process*** | ***Protein Name (HPRD)*** | ***Swiss-Prot ID*** | ***Gene Name*** | ***Molecular Function*** | ***Reg. in SCZ*** | ***SCZ/CTRL ratio*** | ***MW (th)*** | ***pI (th)*** | ***Id. Pept*** | ***% Mas. Matc.*** | ***Spot*** | ***Kruskal-Wallis Analysis*** | ***Other SCZ Studies*** |
| --- | --- | --- | --- | --- | --- | --- | --- | --- | --- | --- | --- | --- | --- |
| Metabolism; Energy pathways | Aconitate hydratase, mitochondrial precursor (Citrate hydro-lyase) (Aconitase) | Q99798 | ACO2 | Catalytic activity | ↑ | 2.17 | 85426.1 | 7.36 | 16 | 39% | S12 | 1.35E-08 | Beasley et al., 2006; Martins-de-Souza et al., 2008. |
|  | Creatine kinase, B chain | P12277 | CKB | Catalytic activity | ↑ | 3.01 | 42644.7 | 5.34 | 10 | 24% | S07 | 7.33E-07 | Prabakaran et al., 2004; Clark et al., 2006; Beasley et al., 2006; Sivagnanasundaram et al., 2007: Martins-de-Souza et al., 2008. |
|  | Dihydrolipoyl dehydrogenase, mitochondrial precursor | P09622 | DLD | Catalytic activity | ↑ | 2.17 | 54150.7 | 7.59 | 14 | 24% | S16 | 9.03E-08 | **No previous reference** |
|  | Dihydropteridine reductase | P09417 | QDPR | Catalytic activity | ↑ | 2.25 | 25803.8 | 6.90 | 11 | 26% | S04 | 8.49E-07 | Prabakaran et al., 2004 |
|  | Gamma enolase (2-phospho-D-glycerate hydro-lyase) (Neuron-specific enolase) | P09104 | ENO2 | Catalytic activity | ↑ | 4.37 | 47137.8 | 4.91 | 18 | 47% | S10 | 2.63E-11 | Vawter et al., 2001; Prabakaran et al., 2004; Sivagnanasundaram et al., 2007; Li et al., 2006; Novikova et al., 2006; Pennington et al., 2007 |
|  | Glyceraldehyde-3-phosphate dehydrogenase | P04406 | GAPDH | Catalytic activity | ↓ | 0.38 | 35922.3 | 8.58 | 7 | 29% | C04 | 6.25E-08 | Prabakaran et al., 2004 |
|  | Phosphoglycerate mutase 1 | P18669 | PGAM1 | Catalytic activity | ↑ | 2.15 | 28673.0 | 6.75 | 13 | 43% | S02 | 4.65E-05 | Prabakaran et al., 2004; Novikova et al., 2006 |
|  | Triosephosphate isomerase | P60174 | TPI1 | Isomerase activity | ↑ | 1.94 | 26538.5 | 6.51 | 8 | 17% | S03 | 0.0001887 | Prabakaran et al., 2004 |
|  | Fructose-bisphosphate aldolase C (Brain-type aldolase) | P09972 | ALDOC | Lyase activity | ↓ | 0.52 | 39325.0 | 6.46 | 16 | 20% | C13 | 0.0005684 | Willson et al., 1980; Johnston-Wilson et al., 2000; Prabakaran et al., 2004; Clark et al., 2006; Novikova et al., 2006; Martins-de-Souza et al., 2008; Martins-de-Souza et al., 2009 |
|  | NADH-ubiquinone oxidoreductase 75 kDa subunit, mitochondrial precursor | P28331 | NDUFS1 | Oxidoreductase activity | ↓ | 0.33 | 79516.3 | 5.89 | 16 | 27% | C12 | 9.12E-05 | Mehler-Wex et al., 2006 |
|  | Peroxiredoxin 6 (Acidic calcium-independent phospholipase A2) | P30041 | **PRDX6** | Peroxidase activity | ↑ | 2.33 | 24904.0 | 6.03 | 13 | 18% | S06 | 0.008882 | **No previous reference** |
|  | Vacuolar ATP synthase catalytic subunit A | P38607 | ATP6V1A | Transporter activity | ↑ | 2.08 | 68178.3 | 5.16 | 12 | 15% | S11 | 0.001956 | Martins-de-Souza et al., 2008. |
|  | ATP synthase alpha chain, mitochondrial precursor (Fragment) | P25705 | ATP5A1 | Transporter activity | ↓ | 3.11 | 59751.1 | 9.16 | 12 | 30% | C11 | 3.67E-06 | Altar et al., 2005; Arion et al., 2007; Martins-de-Souza et al., 2008. |
|  |  |  |  |  |  |  |  |  |  |  |  |  |  |
| Cell growth and/or maintenance | Tubulin beta-5 chain | P07437 | TUBB | Cytoskeletal protein | ↓ | 0.46* | 49671.3 | 4.78 | 14 | 35% | C05/C06/C07 | 1.26E-09 | Beasley et al., 2006 |
|  | Tubulin alpha-1 chain (Alpha-tubulin 1) | P68366 | TUBA1 | Cytoskeletal protein | ↓ | 0.44* | 49924.9 | 4.95 | 14 | 23% | C08/C09 | 5.87E-06 | **No previous reference** |
|  | Tubulin alpha-ubiquitous chain | P68363 | TUBA1B | Cytoskeletal protein | ↓ | 0.46 | 50152.1 | 4.94 | 13 | 36% | C18 | 8.12E-07 | Beasley et al., 2006 |
|  | Tubulin beta-3 chain | Q13509 | TUBB3 | Cytoskeletal protein | ↑ | 9.21 | 50433.2 | 4.83 | 21 | 35% | S09 | 1.74E-09 | **No previous reference** |
|  | Tropomyosin alpha 3 chain (Tropomyosin 3) | P06753 | TPM3 | Cytoskeletal protein binding | ↓ | 0.25 | 32819.1 | 4.68 | 7 | 25% | C02 | 6.45E-05 | **No previous reference** |
|  | Glial fibrillary acidic protein, astrocyte | P14136 | **GFAP** | Structural molecule activity | ↑ | 2.31 | 49880.2 | 5.42 | 7 | 30% | S13 | 2.84E-07 | Johnston-Wilson et al., 2000; Tkachev et al., 2003; Martins-de-Souza et al., 2008. |
|  |  |  |  |  |  |  |  |  |  |  |  |  |  |
| Cell communication ; Signal transduction | Dihydropyrimidinase related protein-2 | Q16555 | DPYSL2 | Cytoskeletal protein binding | ↑ | 1.8 | 62294.2 | 5.95 | 23 | 30% | S08 | 1.02E-08 | Johnston-Wilson et al., 2000; Prabakaran et al., 2004; Sivagnanasundaram et al., 2007; Martins-de-Souza et al., 2008. |
|  | Microtubule-actin crosslinking factor 1, isoform 4 (Fragment) | Q96PK2 | MACF1 | Cytoskeletal protein binding | ↑ | 1.93 | 670156.4 | 5.20 | 19 | 76% | S05 | **1.59** E-12 | **No previous reference** |
|  | Phosphatidylethanolamine-binding protein | P30086 | PEBP-1 | Protease inhibitor activity | ↑ | 1.87 | 20925.8 | 7.43 | 10 | 27% | S01 | 7.13E-05 | Martins-de-Souza et al., 2009 |
|  |  |  |  |  |  |  |  |  |  |  |  |  |  |
| Regulation of gene expression | Breast cancer metastasis-suppressor 1 | Q9HCU9 | BRMS1 | Transcription regulator activity | ↓ | 0.22 | 28460.9 | 4.69 | 11 | 21% | C01 | 0.009254 | **No previous reference** |
|  |  |  |  |  |  |  |  |  |  |  |  |  |  |
| Regulation of nucleobase, nucleoside,  nucleotide and nucleic acid metabolism. | Heterogeneous nuclear ribonucleoproteins C1/C2 | P07910 | HNRPC | RNA binding | ↓ | 0.22 | 33688.3 | 4.95 | 10 | 16% | C03** | 0.001654 | **No previous reference** |
|  |  |  |  |  |  |  |  |  |  |  |  |  |  |
| Transport | Phosphate carrier protein, mitochondrial precursor | Q00325 | SLC25A3 | Transporter activity | ↓ | 0.32 | 40095.2 | 9.45 | 10 | 20% | C14 | 0.0007898 | **No previous reference** |
